# Chronic Human Herpes Virus 8 Infection Successfully Treated With Valganciclovir

**DOI:** 10.1097/WOX.0b013e3181abe7b6

**Published:** 2009-07-15

**Authors:** James J Yun, Veronica Preda, Brad Frankum

**Affiliations:** 1 Campbelltown Hospital, Sydney South West Area Health Service, Campbelltown NSW, Australia; 2 University of Western Sydney, Sydney NSW, Australia; 3 St Vincent's Hospital, Department of Dermatology, University of New South Wales, Sydney, Australia

**Keywords:** human herpes virus 8, valganciclovir, Kaposi sarcoma

## Abstract

**Background**

A 57 year old Italian female with a 3 year history of constituitional symptoms with non-HIV Kaposi sarcoma was referred to the immunology clinic. She was previously diagnosed with chronic human herpes virus 8 (HHV8) infection with positive HHV8 polymerase chain reaction in serum.

**Objective**

Is valganciclovir effective in treating chronic human herpes virus 8 infection without Multicentric Castleman's Disease (MCD)?

**Results**

She was started on valganciclovir, resulting in remission of her symptoms, improvement in inflammatory markers and clearing of detectable HHV8 viraemia

**Conclusion**

Valganciclovir is effective in treating symptomatic HHV8 infections without MCD.

## Introduction

A 57-year-old Italian female with a 3-year history of daily recurrent fever, severe fatigue, arthralgia, and cutaneous Kaposi sarcoma (KS) was referred to the immunology clinic for the management of human herpes virus 8 (HHV8) infection.

## Background History

After a partial gastrectomy for leiomyoma in July 2004, our patient noted daily recurrent fevers up to 38.5°C. This was associated with profound lethargy and severe functional impairment in performing daily living activities. She reported generalized polyarthropathy; however, on clinical examination, there was no evidence of active arthritis. There was no history of weight loss and on examination there was no palpable lymphadenopathy. Extensive investigations were performed including gallium scan, bone scan, CT scan of sinuses, chest, abdomen, and pelvis, and multiple serologies for immunologic and infectious diseases with no definitive diagnosis. She was negative for human immunodeficiency virus (HIV), hepatitis B, and hepatitis C. Autoimmune screens including anti-nuclear antibody, extractable nuclear antigen, and double-stranded DNA, rheumatoid factor, and anti-cyclic citrullinated peptide, anti-neutrophil cytoplasm, and anti-thyroid antibodies were negative. Serum electrophoresis and immunoelectrophoresis showed no paraproteinaemia. Familial Mediterranean fever antibodies were negative. Immunoglobulins and complement levels were normal. Lymphocyte subsets were normal. Endocrine hormone levels were within normal range. Mantoux testing was negative.

Two years after the onset of symptoms, she developed small KS lesions on her fingers, which were biopsy-proven and biopsy-positive for HHV8. Subsequently, our patient had blood samples for quantitative HHV8 to Italy. This confirmed the presence of 80 HHV8 genome equivalents per milliliter of plasma and 2206 HHV8 genome equivalents per 10^6 ^peripheral blood mononuclear cells, using calibrated quantitative real-time polymerase chain reaction (PCR)[1]. Given the blood positivity for HHV8, no oral mucosal or salivary samples were sent for PCR.

In late 2006, 10 cycles of fortnightly cidofovir were commenced. Her fever and constitutional symptoms improved; however, she complained of nausea. Three months after completing cidofovir treatment, her symptoms relapsed and she was referred to the immunology clinic for further management.

On referral to the immunology clinic, repeat blood tests revealed mildly elevated C-reactive protein and erythrocyte sedimentation rate levels and detectable positive HHV8 serology on PCR. A therapeutic trial of acyclovir 200 mg 3 times a day was given with no improvement. Given the significant toxicities associated with cidofovir and foscarnet, alternative therapy was sought. A trial of valganciclovir 900 mg daily was prescribed with symptomatic improvement, reversion to undetectable HHV8 PCR in plasma, and fall in inflammatory markers, and her KS lesions decreased in size from more than 1 cm to a few millimeters in size. She was treated with a trial period of 3 months on valganciclovir and then ceased, with a relapse in her fevers and arthralgias and increase in number of KS lesions including those on the fingers and eyelids. She has recommenced valganciclovir treatment and is receiving another 6 months of therapy. A repeat plasma sample has been tested in our National Infectious Diseases Reference Laboratory while the patient is receiving therapy to monitor response. This is qualitative only using the TaqMan Rapid Testing Probe Assay and it has been negative for HHV8. Clinically, our patient's lesions on her eyelids and fingers have resolved.

Medical history includes obesity, obstructive sleep apnea, hypertension, and multiple surgeries. Regular medications were metoprolol and irbesartan.

## Discussion

Human herpes virus 8 (HHV8) is a member of the gammaherpesvirus subfamily, which also includes Epstein-Barr virus. It is known to cause primary effusion lymphoma, KS, and multicentric Castleman disease (MCD). Chronic HHV8 infection presenting with recurrent fever and constitutional symptoms in immunocompetent patients is rarely reported in the literature. Isolated KS classically is not associated with recurrent fevers or constitutional symptoms. In MCD, patients may present constitutional symptoms but the absence of lymphadenopathy in this patient makes this diagnosis unlikely. Acute primary HHV8 infections may present with transient, nonspecific symptoms including lymphadenopathy, diarrhea, and exanthem, which do not persist for years as in our patient [2].

In 2005, Dagna et al described a case report of a woman who was HIV-negative with a relapsing inflammatory syndrome characterized by fever, lymphadenopathy, splenomegaly, edema, arthrosynovitis, and erythematous rash because of recurrent episodes of HHV8 re-activation[3]. This patient had 4 recurrences that correlated with the rise in HHV8 viral load. Each relapse was treated with either daunorubicin, foscarnet, or cidofovir, which led to suppression of HHV8 viraemia, disappearance of KS, and clinical improvement [3].

In contrast to Dagna et al's case report, our patient had a 3-year history of fever, debilitating fatigue, arthralgia, and KS lesions. Lymphadenopathy, splenomegaly, edema, and rash were absent. Whether our patient had a separate disease entity or symptoms due to chronic HHV8 viraemia along the spectrum of MCD and KS is a point of discussion, as illustrated by the correspondence for the case reported by Dagna et al[4]. Whereas it may be argued that she had a "mild" disease on a spectrum of the HHV8 associated disease because of the absence of other clinical features, the severe debilitating effect of the symptoms on her quality of life must not be underestimated.

Therapeutic management was a challenge in our patient. Initially, she demonstrated a good response to cidofovir. As an ongoing long-term therapeutic option, there were concerns regarding toxicity and ease of administration. Valganciclovir, administered orally, was chosen as an alternative, on the basis of the literature reporting successful reduction in MCD symptoms and HHV8 viraemia[5]. A recent randomized control trial with valganciclovir 900 mg daily in both HIV and non-HIV patients with evidence of HHV8 shedding from their saliva resulted in a significant reduction in HHV8 replication[5]. This supports the demonstrated response in our patient. The resolution of fever and symptomatic improvement with valganciclovir correlated with the suppression of HHV8. The optimal duration of treatment and further management remain unclear at this point. On the basis of the randomized control trial, there was rebound shedding of HHV8 upon cessation of valganciclovir [6].

## Conclusion

We are unaware of other reports of valganciclovir treatment for symptomatic HHV8 infection without MCD, and optimal further management remains to be elucidated.

## Note

Presented at the 2008 ASCIA Annual Scientific Meeting Poster Presentation, November 12-14, Melbourne, Australia.
